# HIV-1 Nef Interacts with LMP7 To Attenuate Immunoproteasome Formation and Major Histocompatibility Complex Class I Antigen Presentation

**DOI:** 10.1128/mBio.02221-19

**Published:** 2020-10-27

**Authors:** Yang Yang, Weiyong Liu, Dan Hu, Rui Su, Man Ji, Yuqing Huang, Muhammad Adnan Shereen, Xiaodi Xu, Zhen Luo, Qi Zhang, Fang Liu, Kailang Wu, Yingle Liu, Jianguo Wu

**Affiliations:** a State Key Laboratory of Virology, College of Life Sciences, Wuhan University, Wuhan, China; b Guangdong Provincial Key Laboratory of Virology, Institute of Medical Microbiology, Jinan University, Guangzhou, China; University of Trento; Albert Einstein College of Medicine

**Keywords:** antigen presentation, negative regulatory factor, Nef, human immunodeficiency virus type 1, HIV-1, immunoproteasome, major histocompatibility complex class I, MHC-I

## Abstract

The proteasome is a major protein degradation machinery with essential and diverse biological functions. Upon induction by cytokines, proteasome subunits β1, β2, and β5 are replaced by β1i/LMP2, β2i/MECL-1, and β5i/LMP7, resulting in the formation of an immunoproteasome (iProteasome). iProteasome-degraded products are loaded onto the major histocompatibility complex class I (MHC-I), regulating immune responses and inducing cytotoxic T lymphocytes (CTLs). Human immunodeficiency virus type 1 (HIV-1) is the causal agent of AIDS. HIV-1-specific CTLs represent a critical immune mechanism limiting viral replication. HIV-1 negative regulatory factor (Nef) counteracts host immunity, particularly the response involving MHC-I/CTL. This study identifies a distinct mechanism by which Nef facilitates immune evasion via suppressing the function of iProteasome and MHC-I. Nef interacts with LMP7 on the endoplasmic reticulum (ER), downregulating the incorporation of LMP7 into iProteasome and thereby attenuating its formation. Moreover, Nef represses the iProteasome function of protein degradation, MHC-I trafficking, and antigen presentation.

## Abstract (precis)

The ubiquitin-proteasome system (UPS) is essential for the degradation of damaged proteins, which takes place in the proteasome. Upon activation by cytokines, the catalytic subunits of the proteasome are replaced by distinct isoforms resulting in the formation of an immunoproteasome (iProteasome). iProteasome generates peptides used by major histocompatibility complex class I (MHC-I) for antigen presentation and is essential for immune responses. HIV-1 is the causative agent of AIDS, and HIV-1-specific cytotoxic T lymphocytes (CTLs) provide immune responses limiting viral replication. This study identifies a distinct mechanism by which HIV-1 promotes immune evasion. The viral protein negative regulatory factor (Nef) interacts with a component of iProteasome, LMP7, attenuating iProteasome formation and protein degradation function, and thus repressing the MHC-I antigen presentation activity of MHC-I. Therefore, HIV-1 targets LMP7 to inhibit iProteasome activation, and LMP7 may be used as the target for the development of anti-HIV-1/AIDS therapy.

## INTRODUCTION

Proteasome, a multiprotein complex essential for protein degradation, performs important functions, such as the clearance of mutated or misfolded proteins, cell signaling, and antigen presentation (1, 2). The 20S core particle of the constitutive proteasome (cProteasome) has a barrel-shaped structure comprising four stacked rings; the two outer rings contain α subunits and act as binding sites for complexes regulating the access of proteins into the proteasome inner chamber, and the two inner rings contain β subunits. They include β1, β2, and β5 active sites referred to “caspase-,” “trypsin-,” and “chymotrypsin-like,” respectively (3). Upon cytokine stress, such as interferon (IFN) activation, the three catalytic subunits are replaced by distinct isoforms, namely, β1i (LMP2), β2i (MECL-1), and β5i (LMP7), forming an immunoproteasome (iProteasome) (4, 5). For the assembly of iProteasome, full-length low-molecular-mass protein 7 (proLMP7) is cleaved to generate a mature LMP7 (matLMP7) that represents is a key factor in iProteasome formation (6, 7). Mapping of LMP2 and LMP7 to the major histocompatibility complex class I (MHC-I) locus, together with the IFN-induced activation prompted the hypothesis that iProteasome has a role in generating peptides for MHC-I antigen presentation (5).

MHC-I is an efficient surveillance system that recognizes and presents antigens when the host is invaded by pathogens. A critical step in the MHC-I pathway consists of processing antigens into smaller peptides and translocating them onto a peptide loading complex (PLC) (8–11). MHC-I presents a diverse array of antigenic peptides, known as the immunopeptidome, to circulating cytotoxic T lymphocytes (CTLs) (11, 12). The antigen processing pathway executes a series of steps to ensure the assembly of peptides and MHC-I in the endoplasmic reticulum (ER) (13).

Upon infecting the host, viruses reproduce inside living cells and must counteract and evade the immune defense. Human immunodeficiency virus type 1 (HIV-1) is the causal agent of AIDS (14, 15). Activation of HIV-1-specific CTLs represents the critical immune response limiting viral replication (16–18), and MHC-I variants determine the disease progression of AIDS (19, 20). The HIV-1 genome encodes 5 proteins essential for viral replication and 4 accessory proteins (21, 22). One of the accessory proteins, the negative regulatory factor (Nef), counteracts host immunity by interacting with phosphofurin acidic cluster sorting protein-1 (PACS-1) and phosphatidylinositol 3-kinase (PI3K). These interactions attenuate the translocation of MHC-I to the *trans*-Golgi network (23), downregulate CD4 by hijacking AP-2 (24), enhance viral infectivity by excluding SERINC3/5 from the virions (25, 26), and induce secretion of exosomes from infected cells, stimulating viral spread (27). Importantly, Nef is a multifunctional protein, and several additional functions have been ascribed to this molecule.

The present study identifies a distinct pathway used by HIV-1 to evade immune responses, which involves the attenuation of the functions of the immunoproteasome and MHC-I. In this mechanism, Nef interacts with LMP7 on the ER membrane and downregulates the incorporation of LMP7 into the iProteasome, reducing iProteasome formation. Moreover, Nef represses the protein degradation function of iProteasome and inhibits MHC-I trafficking and antigen presentation activity.

## RESULTS

### LMP7 is associated with Nef.

**FIG 1:**
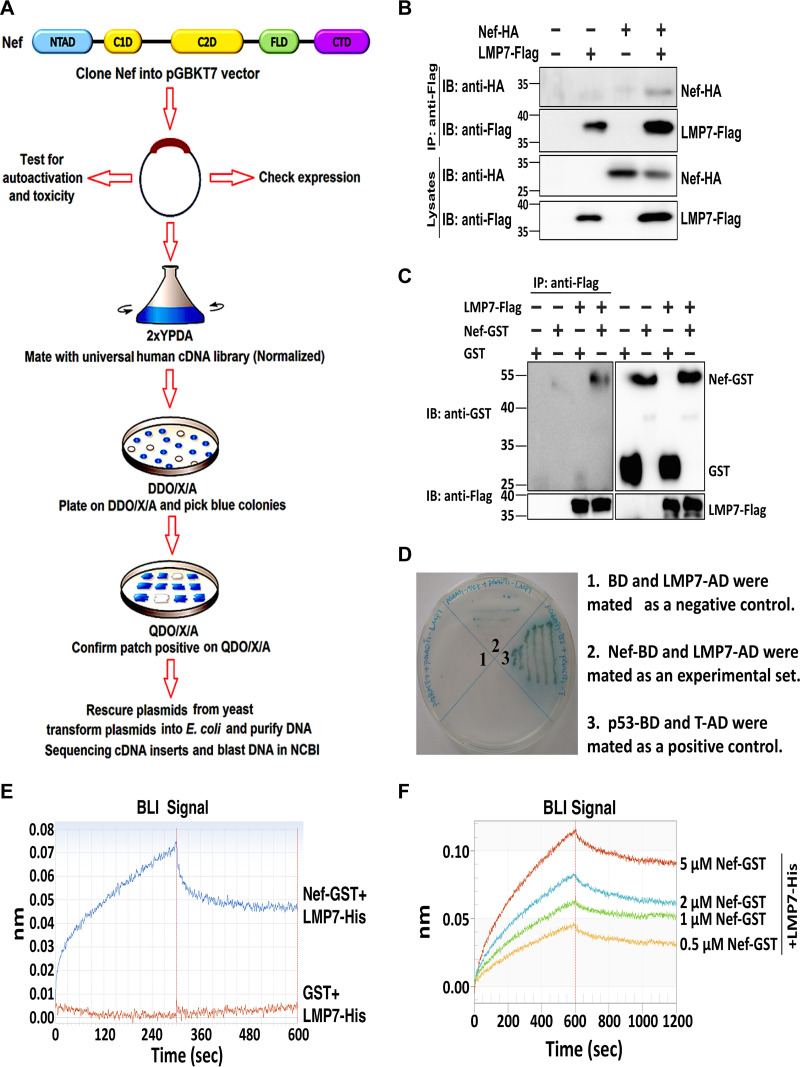
LMP7 interacts with Nef. (A) Flow chart of yeast two-hybrid system to screen proteins interacting with Nef. NTAD, N-terminal anchor domain; C1D, core 1 domain; C2D, core 2 domain; FLD, flexible loop domain; CTD, C-terminal domain. (B) 293T cells were transfected with pNef-HA and pLMP7-Flag for 24 h, and cell lysates were prepared for coimmunoprecipitation (co-IP). Anti-Flag antibodies were used for co-IP, and anti-HA or anti-Flag antibodies were used to detect Nef-HA or LMP7-Flag. Cell lysates were detected with anti-HA and anti-Flag antibodies. (C) Purified LMP7-His-Flag protein or no protein was mixed with GST or Nef-GST. Proteins were pulled down by anti-Flag antibodies (IP), and detected with anti-GST antibodies (IB). Proteins in cell lysates were identified with anti-GST and anti-Flag antibodies. (D) The yeast two-hybrid system was used to confirm the interaction between Nef and LMP7. Mated yeast cells of Nef fused with Gal4 DNA-binding domain (BD) and LMP7 fused with the Gal4 activation domain (AD). BD and LMP7-AD were mated as a negative control using agar containing QDO (−Ade/−His/−Leu/−Trp) and X-α-Gal (area 1). Nef-BD and LMP7-AD were mated as the experimental set (area 2). p53-BD and T-AD were mated as a positive control (area 3). (E) Biolayer interferometry (BLI) wavelength shift of Nef-GST with LMP7-His (blue) and GST with LMP7-His (red) was recorded during the association and dissociation of these molecules. (F) BLI wavelength shift of increasing concentrations of Nef-GST with a constant of LMP7-His was recorded during the association and dissociation of these molecules. The results shown are the representative of three independent experiments.

Nef is a critical protein necessary for HIV-1 pathogenesis, immune evasion, and viral spread. To determine the mechanism by which Nef regulates the immune response, we initially screened proteins interacting with Nef by utilizing a yeast two-hybrid system (Nef used throughout this study corresponds to the NL4-3 variant of HIV-1) (Fig. 1A). The results documented that LMP7 is associated with Nef. Coimmunoprecipitation (co-IP) indicated that Nef interacts with LMP7 in human embryonic kidney (HEK)293T cells (Fig. 1B). A protein-protein pulldown assay revealed that the purified complex of Nef and glutathione *S*-transferase (Nef-GST) directly interacts with LMP7 (Fig. 1C). A yeast two-hybrid experiment further demonstrated that Nef could associate with LMP7 (Fig. 1D). The strength of the interaction between LMP7 and Nef was determined by BioLayer interferometry (BLI). The association of LMP7 with Nef was robust, as indicated by the significantly higher value of wavelength shift for LMP7 and Nef-GST (71.1 pm) than that for LMP7 and GST (0.7 pm) (Fig. 1E). This finding confirmed that LMP7 is tightly associated with Nef. Additionally, a series of concentrations of Nef protein was analyzed in the presence of the same amount of the LMP7 protein; the wavelength shift was increased at higher Nef concentrations, and the binding affinity of LMP7 to Nef expressed as the dissociation constant (*K_d_*) value was high at 734.7 nM (Fig. 1F).

### LMP7 binds to Nef through the sequence from amino acids 69 to 160.

**FIG 2:**
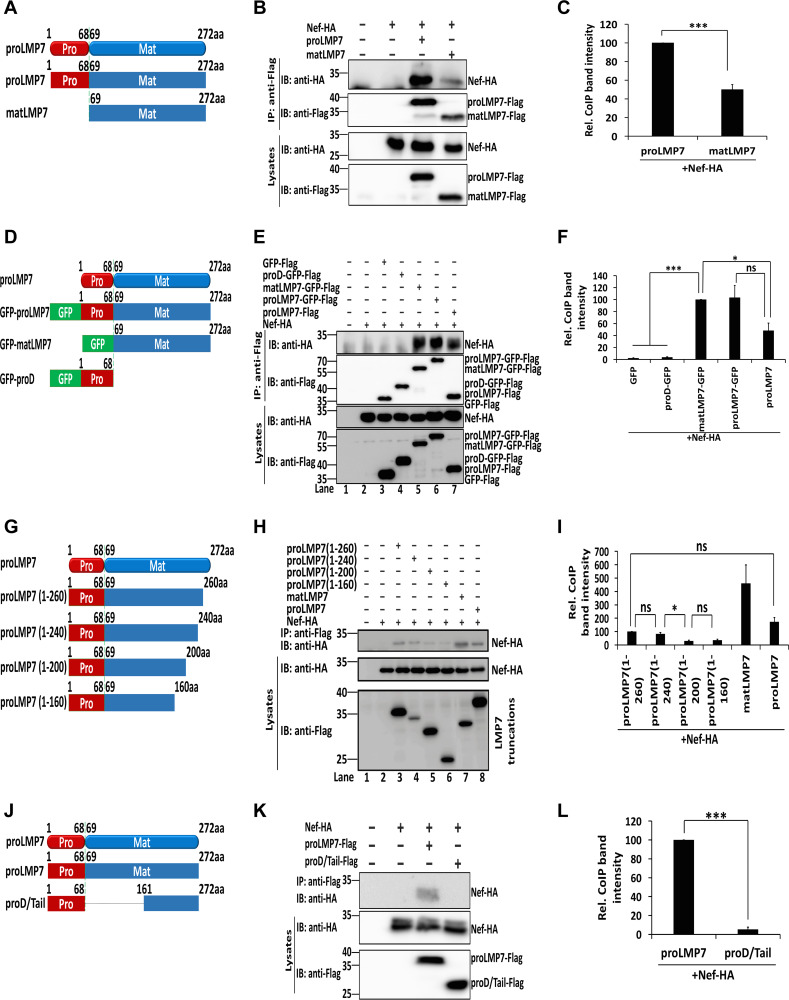
LMP7 interacts with Nef by sequences from amino acids 69 to 160. (A) Schematic structure of proLMP7 and matLMP7. (B, C) 293T cells were cotransfected with pNef-HA and pproLMP7-Flag or pmatLMP7-Flag for 24 h. Cell lysates were prepared for co-IP (B). Relative co-IP band intensity reflects the ratio of pulldown Nef-HA to Nef-HA and proLMP7-Flag or matLMP7-Flag in the lysates. The relative intensity of the co-IP band of Nef-HA + proLMP7-Flag was set as 100% (C). (D) Schematic structure of fusion proteins proLMP7-GFP, matLMP7-GFP, and proD-GFP. (E, F) 293T cells were cotransfected with p-Nef-HA and p-GFP-Flag, p-proD-GFP-Flag, p-matLMP7-GFP-Flag, p-proLMP7-GFP-Flag, or p-proLMP7-Flag. After 24 h, cell lysates were prepared for co-IP (E). Relative co-IP band intensity reflects the ratio of pulldown Nef-HA to Nef-HA and LMP7-Flag truncations in the lysates. The relative intensity of the co-IP band of Nef-HA + matLMP7-GFP was set as 100% (F). (G) Schematic structure of truncated LMP7. (H, I) 293T cells were cotransfected with pNef-HA and p-proLMP7(1 to 260)-Flag, p-proLMP7(1 to 240)-Flag, p-proLMP7(1 to 200)-Flag, p-proLMP7(1 to 160)-Flag, p-matLMP7-Flag, or pLMP7-Flag. After 24 h, cell lysates were prepared for co-IP (H). The relative co-IP band intensity reflects the ratio of pulldown Nef-HA to Nef-HA and LMP7-Flag truncations in the lysates. The relative intensity of the co-IP band of Nef-HA + proLMP7(1 to 260) was set as 100% (I). (J) Schematic structure of proLMP7 and proD/Tail. (K, L) 293T cells were cotransfected with pNef-HA and p-proLMP7-Flag or p-proD/Tail-Flag. After 24 h, cell lysates were prepared for co-IP (K). The relative co-IP band intensity reflects the ratio of pulldown Nef-HA to Nef-HA and LMP7-Flag truncations in the lysates. The relative intensity of the co-IP band of Nef-HA + proLMP7 was set as 100% (L). (B, E, H, K) Anti-Flag antibodies were used for IP and anti-HA/anti-Flag or anti-HA antibodies were used for IB. Cell lysates were detected with anti-HA and anti-Flag antibodies. The results shown are the representative of three independent experiments. The quantitative data represent the mean and standard deviation of three independent experiments. ns, not significant; *, *P* ≤ 0.05; **, *P* ≤ 0.01; ***, *P* ≤ 0.001.

Upon IFN-γ induction, full-length low-molecular-mass protein 7 LMP7 (proLMP7) is cleaved into a mature LMP7 (matLMP7) before iProteasome assembly. To evaluate the requirement of the two forms of LMP7 in the interaction with Nef, plasmids expressing proLMP7 and matLMP7 were constructed (Fig. 2A). Both proLMP7 and matLMP7 were able to pull down Nef (Fig. 2B), indicating that matLMP7 is sufficient for the interaction. However, the full-length LMP7 was able to pull down more Nef than matLMP7 (Fig. 2C). Next, three fusion proteins were generated in which the green fluorescent protein (GFP) was fused to proLMP7, matLMP7, and the prodomain of LMP7 (proD) (Fig. 2D). proLMP7, proLMP7-GFP, and matLMP7-GFP pulled down Nef, but proD-GFP did not (Fig. 2E), suggesting that the prodomain is not sufficient to mediate interaction with Nef. The GFP-fused proLMP7 and matLMP7 pulled down a comparable amount of Nef, but both constructs pulled down a larger quantity of Nef than proLMP7; the difference between GFP-fused proLMP7 and proLMP7 was not significant (Fig. 2F).

To narrow down the amino acid sequences interacting with LMP7, 4 deletions were generated in the LMP7 molecule (Fig. 2G). Nef interacted strongly with LMP7 and LMP7(amino acids 69 to 272), moderately with LMP7(1 to 260) and LMP7(1 to 240), and weakly with LMP7(1 to 200) and LMP7(1 to 160) (Fig. 2G to I). These findings indicate that amino acids from 200 to 240 are required for efficient interaction with Nef.

Subsequently, we tested an LMP7 truncation without the domain from 69 to 160 residues (Fig. 2J), which showed that this truncation proD/Tail failed to interact with Nef, implying that the region corresponding to amino acids 69 to 160 is crucial for the binding with Nef (Fig. 2K and L). Together, these results suggest that the LMP7 domains comprising amino acid residues 69 to 160 and 200 to 240 are essential for the interaction between LMP7 and Nef; however, the prodomain (amino acids 1 to 68) is dispensable for this interaction.

### Nef interacts with LMP7 via the C2D domain.

**FIG 3:**
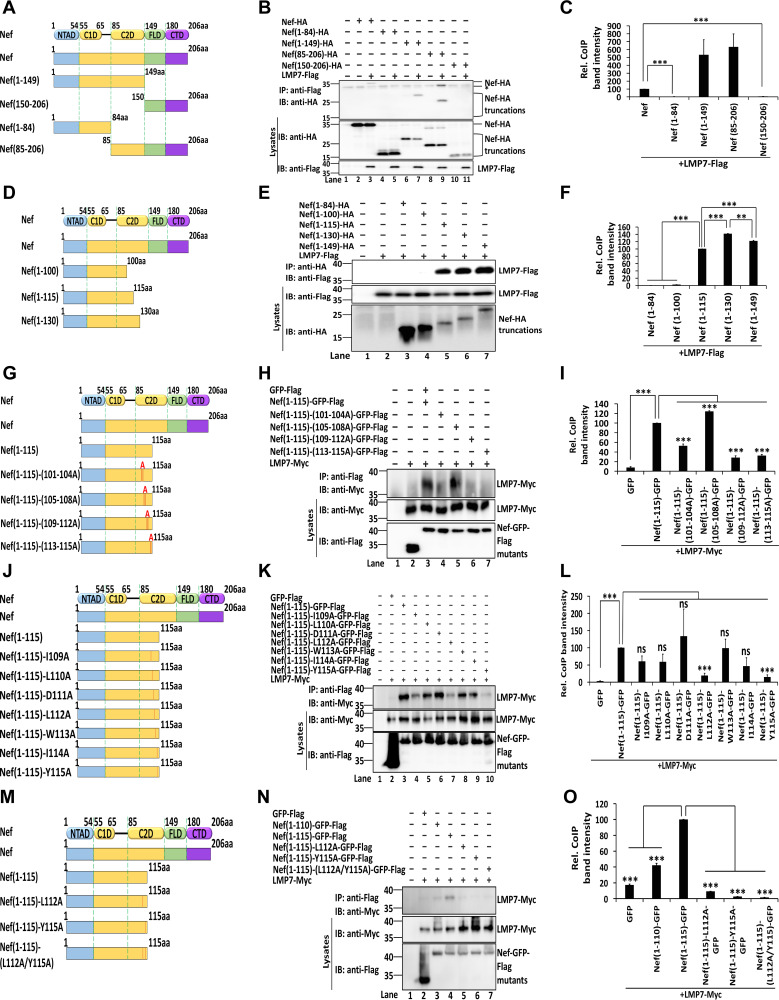
Nef interacts with LMP7 through the C2D domain. (A) Schematic structure of Nef and truncated Nef. NTAD, N-terminal anchor domain; C1D, core 1 domain; C2D, core 2 domain; FLD, flexible loop domain; CTD, C-terminal domain. (B, C) 293T cells were cotransfected with pLMP7-Flag and pNef-HA, pNef(1 to 84)-HA, pNef(1 to 149)-HA, pNef(85 to 206)-HA, or pNef(150 to 206)-HA for 24 h. The relative co-IP band intensity reflects the ratio of pulldown Nef-HA or Nef-HA truncations to Nef-HA or Nef-HA truncations and LMP7-Flag in lysates. The relative intensity of the co-IP band of Nef-HA + LMP7-Flag was set as 100% (C). (D) Schematic structure of Nef truncations within amino acids 85 to 149. (E, F) 293T cells were cotransfected with pLMP7-Flag and pNef(1 to 84)-HA, pNef(1 to 100)-HA, pNef(1 to 115)-HA, pNef(1 to 130)-HA, or pNef(1 to 149)-HA for 24 h. Relative co-IP band intensity reflects the ratio of pulldown Nef-HA truncations to Nef-HA truncations and LMP7-Flag in lysates. The relative intensity of the co-IP band of Nef(1 to 84) + LMP7-Flag was set as 100% (F). (B, E) Cell lysates were prepared for co-IP. Anti-Flag antibodies were used for IP and detected with anti-HA antibodies. Cell lysates were detected with anti-HA and anti-Flag antibodies. (G) Schematic structure of Nef point mutations, in which 3 or 4 residues in Nef(1 to 115) were replaced by alanine. (H, I) 293T cells were cotransfected with pLMP7-Myc and pGFP-Flag, pNef(1 to 115)-GFP-Flag, pNef(1 to 115)-(101-104A)-GFP-Flag, pNef(1 to 115)-(105-108A)-GFP-Flag, pNef(1 to 115)-(109-112A)-GFP-Flag, or pNef(1 to 115)-(113-115A)-GFP-Flag for 24 h. The relative co-IP band intensity reflects the ratio of pulldown LMP7-Myc to Nef-Flag mutants and LMP7-Myc in lysates. The relative intensity of the co-IP band of Nef(1 to 115)-GFP + LMP7-Myc was set as 100% (I). (J) Schematic structure of seven single-residue mutations in Nef, in which residues were mutated individually to alanine in Nef(1 to 115). (K, L) 293T cells were cotransfected with pLMP7-Myc and pGFP-Flag, pNef(1 to 115)-GFP-Flag, pNef(1 to 115)-(I109A)-GFP-Flag, pNef(1 to 115)-(L110A)-GFP-Flag, pNef(1 to 115)-(D111A)-GFP-Flag, pNef(1 to 115)-(L112A)-GFP-Flag, pNef(1 to 115)-(W113A)-GFP-Flag, pNef(1 to 115)-(I114A)-GFP-Flag, or pNef(1 to 115)-(Y115A)-GFP-Flag for 24 h. The relative co-IP band intensity reflects the ratio of pulldown LMP7-Myc to Nef-GFP-Flag mutants and LMP7-Myc in lysates. The intensity of the co-IP band intensity of Nef(1 to 115)-GFP + LMP7-Myc was set as 100% (L). (M) Schematic structure of two single-residue and one double-residue mutations in Nef. (N, O) 293T cells were cotransfected with pLMP7-Myc and pGFP-Flag, pNef(1 to 110)-Flag, pNef (1 to 115)-Flag, pNef(1 to 115)-(L112A)-Flag, pNef(1 to 115)-(Y115A)-Flag, or pNef(1 to 115)-(L112A/Y115A)-Flag for 24 h. The relative co-IP band intensity reflects the ratio of pulldown LMP7-Myc to Nef-GFP-Flag mutants and LMP7-Myc in lysates. The relative intensity of the co-IP band of Nef(1 to 115)-GFP + LMP7-Myc was set as 100% (O). (H, K, N) Cell lysates were prepared for co-IP. Anti-Flag antibodies were used for IP and detected with anti-Myc antibodies. Cell lysates were detected with anti-Myc and anti-Flag antibodies. The results shown are representative of three independent experiments. The quantitative data represent the mean and standard deviation of three independent experiments. ns, not significant; *, *P* ≤ 0.05; ** *P* ≤ 0.01; *** *P* ≤ 0.001.

The domains of Nef interacting with LMP7 were also determined. For this purpose, 4 truncated mutants of Nef were constructed (Fig. 3A). LMP7 interacted with Nef, Nef(1 to 149), and Nef(85 to 206), but failed to interact with Nef(1 to 84) and Nef(150 to 206) (Fig. 3B and C). Thus, amino acids 85 to 149 of Nef are essential for the interaction with LMP7. Next, 3 truncations of Nef within amino acids 85 to 149 were constructed (Fig. 3D). LMP7 interacted with Nef(1 to 115), Nef(1 to 130), and Nef(1 to 149), but not with Nef(1 to 84) and Nef(1 to 100) (Fig. 3E), demonstrating that residues from 101 to 115 of Nef are required for its interaction with LMP7. Also, we observed that the strength of the interaction between Nef(1 to 130) and LMP7 was approximately 40% higher than that of Nef(1 to 115), while the interaction between Nef(1 to 130) and LMP7 was approximately 20% stronger than that of Nef(1 to 149) (Fig. 3F). The residues of Nef essential for interacting with LMP7 were determined by generating four site-directed mutations (Fig. 3G). LMP7 interacted strongly with Nef(1 to 115) and Nef(1 to 115)-(105 to 108A), weakly with Nef (1 to 115)-(101 to 104A), and did not interact with GFP, Nef(1 to 115)-(109 to 112A), or Nef(1 to 115)-(113 to 115A) (Fig. 3H and I). These results indicate that amino acid residues from 109 to 115 are crucial for the interaction between Nef and LMP7. Each of the 7 residues was then separately mutated to alanine in Nef(1 to 115) (Fig. 3J). LMP7 interacted strongly with Nef(1 to 115), Nef(1 to 115)-(I109A), Nef(1 to 115)-(L110A), Nef(1 to 115)-(D111A), Nef(1 to 15)-(W113A), and Nef(1 to 115)-(I114A); weakly with Nef(1 to 115)-(L112A); and minimally with Nef(1 to 115)-(Y115A) (Fig. 3K). Moreover, in comparison with Nef(1 to 115), the strength of the interaction between LMP7 and Nef(1 to 115)-(L112A) or Nef(1 to 115)-(Y115A) was significantly reduced to approximately 19% and 14%, respectively (Fig. 3L), suggesting that residues 112 and 115 are the most important for the interaction. Interestingly, it has been known that residues L112 and Y115 are relatively conserved in primary HIV-1 isolates and have an essential function in the oligomerization of Nef (28, 29).

Finally, the role of L112 and Y115 in the interaction with LMP7 was assessed using a double-residue mutant (Fig. 3M). LMP7 interacted with Nef(1 to 110) and Nef(1 to 115), interacted weakly with Nef(1 to 115)-(L112A), and minimally interacted with Nef(1 to 115)-(Y115A), but failed to interact with Nef(1 to 115)-(L112A/Y115A) (Fig. 3N). Moreover, in comparison with Nef(1 to 115), the strength of the interaction between LMP7 and Nef(1 to 115)-(L112A/Y115A) was significantly reduced to approximately 1.4% (Fig. 3O), demonstrating that both L112 and Y115 within the C2D domain of Nef are essential for its interaction with LMP7. Several other amino acid residues within the C2D domain have been documented as necessary for Nef oligomerization and interaction with host proteins (28, 30, 31). Here, we documented that residues L112 and Y115 within this domain are required for interacting with LMP7.

### Nef interacts with LMP7, attenuating immunoproteasome formation.

**FIG 4:**
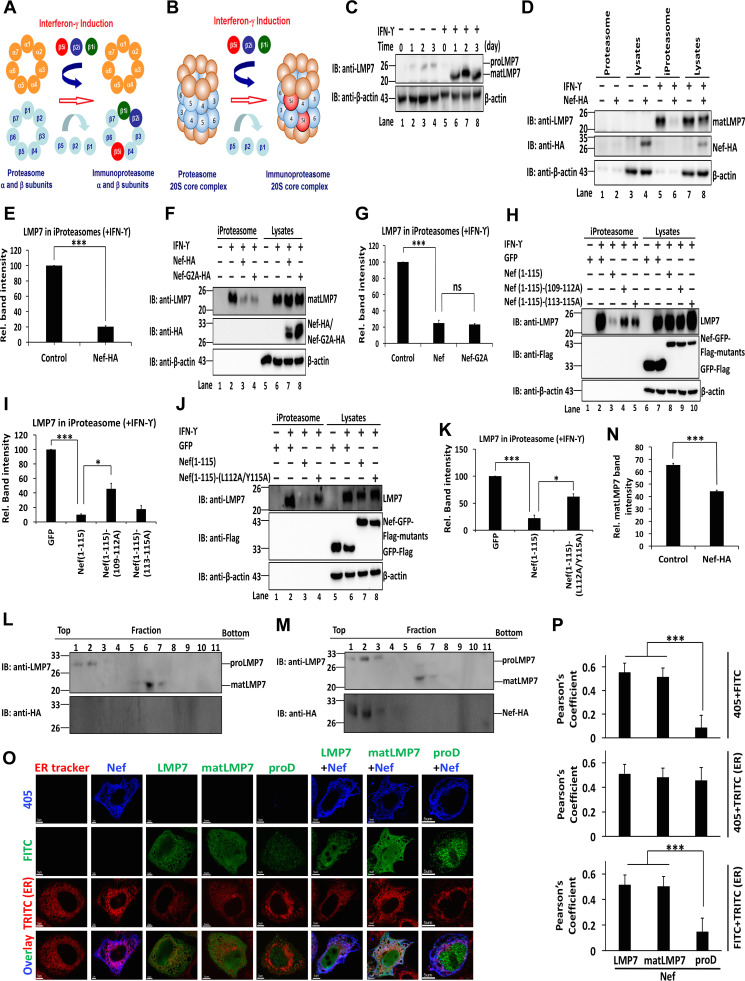
Nef interacts with LMP7 to attenuate immunoproteasome formation. (A) Schematic structure of α and β rings of the cProteasome and iProteasome. Upon IFN-γ stimulation, immunoproteasome-specific subunits β1i, β2i, and β5i are assembled into the iProteasome. (B) Schematic structure of 20S core particles of proteasome and iProteasome. Upon IFN-γ stimulation, immunoproteasome specific subunits β1i, β2i, and β5i are assembled into the iProteasome. (C) HeLa cells were treated with rhIFN-γ for 0, 1, 2, and 3 days and LMP7 and β-actin were determined by Western blotting. (D, E) Lysates, proteasomes, and iProteasomes were prepared from HeLa cells transfected with an empty vector or pNef-HA for 24 h and then cultured in the presence or absence of rhIFN-γ for 24 h. LMP7, Nef, and β-actin were detected by Western blotting (D). Relative band intensity reflects the ratio of LMP7 in iProteasomes to LMP7 in the lysates. The relative intensity of the band corresponding to the empty vector (negative control) was set as 100% (E). (F, G) Lysates, proteasomes, and iProteasomes were prepared from HeLa cells transfected with empty vector, pNef-HA, or pNef-G2A-HA for 24 h and then cultured in the presence or absence of rhIFN-γ for 24 h. LMP7, Nef, Nef-G2A, and β-actin were detected by Western blotting (F). Relative band intensity stands for the ratio of LMP7 in immunoproteasomes to LMP7 in lysates. The relative intensity of band corresponding to the empty vector (negative control) was set as 100% (G). (H, I) Lysates, proteasomes, and iProteasomes were prepared from HeLa cells transfected with pGFP-Flag, pNef(1 to 115)-Flag, pNef(11 to 115)-(109-112A)-Flag, or pNef(11 to 115)-(113-115A)-Flag for 24 h and then cultured in the presence or absence of rhIFN-γ for 24 h. LMP7, GFP, Nef(11 to 115), Nef (11 to 115)-(109-112A), or Nef (11 to 115)-(113-115A) and β-actin were detected by Western blotting (H). Relative band intensity stands for the ratio of LMP7 in immunoproteasomes to LMP7 in lysates. The relative intensity of the band corresponding to pGFP-Flag (negative control) was set as 100% (I). (J, K) Lysates, proteasomes, and iProteasomes were prepared from HeLa cells transfected with pGFP-Flag, pNef(11 to 115)-Flag, or pNef (11 to 115)-(L112A/Y115A) for 24 h and then cultured in the presence or absence of rhIFN-γ for 24 h. LMP7, GFP, Nef(11 to 115), Nef(11 to 115)-(L112A/Y115A), and β-actin were detected by Western blotting (J). Relative band intensity reflects the ratio of LMP7 in iProteasomes to LMP7 in lysates. The relative intensity of the band of pGFP-Flag (negative control) was set as 100% (K). (L, M, N) HeLa cells were transfected with an empty vector (L) or pNef-HA (M) for 24 h and then cultured in the presence or absence of rhIFN-γ for 24 h. Subsequently, 11 fractions of cell lysates, from top to bottom, were collected after ultracentrifugation. The levels of proLMP7, matLMP7, and Nef were determined by Western blotting. Relative matLMP7 band intensity reflects the ratio of matLMP7 in all fractions to the sum of proLMP7 and matLMP7 in all fractions. The relative intensity of the matLMP7 band in cells treated with the empty vector served as a negative control (N). (O, P) HeLa cells were transfected with empty vector, pNef-HA, pLMP7-Flag, p-matLMP7-Flag, p-proD-Flag, pNef-HA+pLMP7-Flag, pNef-HA+p-matLMP7-Flag, or pNef-HA+p-proD-Flag for 24 h. Transfected cells were stained with ER-Tracker (red), anti-HA antibodies (blue), and anti-Flag antibodies (green). Pictures were taken using the FluoView FV1000 (Olympus) confocal microscope. Pearson’s coefficient values were calculated using the Olympus Fluoview Viewer, v.1.7a (P). The results shown are representative of three independent experiments. The quantitative data represent the mean and standard deviation of three independent experiments. ns, not significant; *, *P* ≤ 0.05; **P ≤ 0.01; ***, *P* ≤ 0.001.

It has been shown that LMP7 is one of the crucial components involved in the formation of iProteasome upon the induction by IFN-γ. Since the biological effect of the interaction between Nef and LMP7 has been demonstrated and LMP7 is a key factor in the assembly of iProteasome (Fig. 4A and B) (32–34), it is reasonable to raise the possibility that Nef may affect iProteasome formation by interaction with LMP7. To verify this hypothesis, HeLa cells were treated with recombinant human IFN-γ (rhIFN-γ). proLMP7 was present in the absence of rhIFN-γ but was not detected in the presence of rhIFN-γ; in contrast, matLMP7 was not detected in the absence of rhIFN-γ but became undetectable after the treatment with rhIFN-γ (Fig. 4C). These results indicate that IFN-γ induces LMP7 maturation. Cell lysates, proteasomes, and iProteasomes were then prepared from cells transfected with Nef and treated with rhIFN-γ. The successful purification of proteasomes was demonstrated by the absence of β-actin in proteasomes or iProteasomes, while this protein was present in cell lysates (Fig. 4D) (35). Endogenous LMP7 was not detected in proteasomes and cell lysates in the absence of rhIFN-γ but was detected in iProteasomes and lysates of HeLa cells stimulated by rhIFN-γ (Fig. 4D). In the presence of rhIFN-γ, Nef was not detected in the iProteasome but was identified in cell lysates (Fig. 4D), suggesting that Nef was not associated with the fully assembled iProteasome. Interestingly, the level of LMP7 was significantly attenuated by Nef in iProteasome, but remained relatively unaffected in cell lysate (Fig. 4D). Moreover, the level of LMP7 in iProteasomes was reduced by 80% in the presence of Nef (Fig. 4E), suggesting that Nef attenuated the incorporation of LMP7 into iProteasome and thereby downregulated iProteasome formation.

To determine the impact of Nef myristoylation on the formation of iProteasomes, the second amino acid of Nef glycine, which is critical for the myristoylation of Nef (36), was mutated to alanine (Nef-G2A). Intriguingly, in comparison with samples transfected with the empty vector, the concentration of LMP7 in iProteasomes was markedly decreased when Nef or Nef-G2A were expressed, and upon treatment of cells with rhIFN-γ, even the expression of LMP7 in lysates of both Nef- and Nef-G2A-transfected cells was higher than that in cells transfected with the empty vector (Fig. 4F). Additionally, both Nef and Nef-G2A reduced the level of LMP7 in iProteasomes by 80% in the presence of rhIFN-γ (Fig. 4G). Together, these data document that the myristoylation of Nef is not related to the formation of iProteasome.

To assess the effect of the association of Nef and LMP7 on the assembly of iProteasomes, three Nef mutants were tested. The content of LMP7 in iProteasomes was highly attenuated by Nef(1 to 115), while the impact of both Nef(1 to 115)-(109 to 112A) and Nef(1 to 115)-(113 to 115A) (Fig. 4H) was markedly less pronounced. The level of LMP7 in iProteasomes was reduced by approximately 90% in the presence of Nef, by 60% in the presence of Nef(1 to 115)-(109 to 112A), and by 80% in the presence of Nef(1 to 115)-(103 to 115A) (Fig. 4I), suggesting that residues from 109 to 115 on Nef are critical for the inhibition of iProteasome formation. An iProteasome formation experiment was performed next to determine the effect the double-residue mutant of Nef on the inhibition of iProteasome formation. The data showed that the level of LMP7 was significantly reduced by Nef(1 to 115) in iProteasomes, whereas LMP7 was reduced slightly by Nef(1 to 115)-(L112A/Y115A) in iProteasomes (Fig. 4J). LMP7 in iProteasomes was significantly reduced by 80% in the presence of Nef and by only 40% in the presence of Nef(1 to 115)-(L112A/Y115A) (Fig. 4K). These results imply that residues L112 and Y115 of Nef are important for the attenuation of iProteasome assembly.

The role of Nef in the regulation of iProteasome was further analyzed using gradient ultracentrifugation. In the absence of Nef, proLMP7 was detected in the top fractions 1 and 2, while matLMP7 was distributed in the bottom fractions 6 and 7 (Fig. 4L). In the presence of Nef, both Nef and proLMP7 were detected in the top fractions 1 to 3, and matLMP7 was present in the bottom fractions 6 and 7 (Fig. 4M). These results confirm that Nef is not associated with the assembled iProteasome but instead it interacts with LMP7, preventing the incorporation of LMP7 into iProteasome. Additionally, Nef inhibits LMP7 already incorporated into iProteasome (Fig. 4N), which is consistent with the data in Fig. 4D.

The critical steps of iProteasome formation take place on the ER membrane (37). Our data demonstrated that both Nef and LMP7, whether expressed alone or together, were distributed mostly on the ER membrane (Fig. 4O and P). Interestingly, Nef and matLMP7 were also colocalized and distributed mostly on the ER membrane. However, Nef did not colocalize with proD, which was not associated with the ER membrane, suggesting that the interaction between Nef and LMP7 is necessary for the colocalization on the ER membrane (Fig. 4O and P). Thus, a relatively large amount of Nef interacts with LMP7 on the ER membrane, preventing LMP7 from incorporating into the iProteasome and thereby attenuating the assembly of iProteasome formation at an early stage.

### Nef attenuates protein degradation activity and MHC-I antigen presentation function.

**FIG 5:**
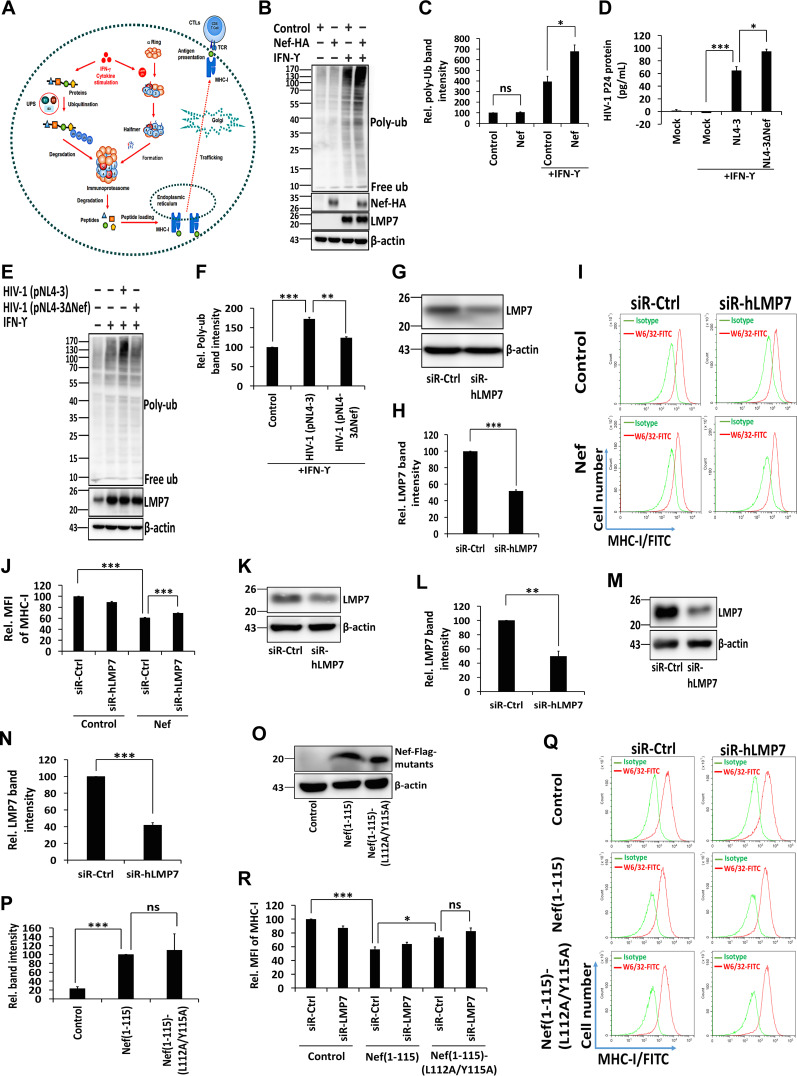
Nef attenuates the protein degradation activity of iProteasome and MHC-I antigen presentation function. (A) Diagram of UPS, iProteasome, MHC-I, and CTL. (B, C) HeLa cells were transfected with pNef for 24 h and then cultured in the presence or absence of rhIFN-γ. The ubiquitination of proteins, Nef-HA, and the expression of LMP7 and β-actin were determined by Western blotting (B). Relative poly-ubiquitin (Ub) band intensity reflects the ratio of the ubiquitination of proteins to the β-actin. The relative intensity of the poly-Ub band of cells transfected with the empty vector untreated with rhIFN-γ (negative control) was set as 100% (C). (D to F) CD4^+^ Jurkat T cells were infected with HIV-1 (pNL4-3) or Nef-deficient HIV-1 (pNL4-3dNef) at the MOI of 10 ng p24/10^6^ cells and treated with rhIFN-γ. The concentration of the p24 protein in cell lysates was determined by ELISA (D). The ubiquitination of proteins and expression of LMP7 and β-actin were determined by Western blotting (E). Relative poly-Ub band intensity represents the ratio of the ubiquitination of proteins to the corresponding β-actin. The relative intensity of the poly-Ub band of uninfected cells treated with rhIFN-γ (negative control) was set as 100% (F). (G, H) CD4^+^ Jurkat T cells were transfected with siR-Ctrl or siR-hLMP7 for 48 h. Cell lysates were prepared, and the levels of LMP7 and β-actin were measured by Western blotting (G). The intensity of protein bands was quantified; relative LMP7 band intensity reflects the ratio of LMP7 to β-actin (H). (I, J) pLenti-Flag and pLenti-Nef-Flag CD4^+^ Jurkat T cells were transfected with siR-Ctrl or siR-hLMP7 for 48 h. Cells were collected and analyzed by flow cytometry after staining with the W6/32 anti-MHC-I-fluorescein isothiocyanate (FITC) antibody. Cells stained with an IgG isotype antibody served as an MHC-I negative control (I). Mean fluorescence intensity (MFI) was calculated for all W6/32 MHC-I-FITC-stained cells (J). (K to N) CD4^+^ Jurkat T cells infected with pLenti-Nef(1 to 115)-Flag (K, L) or pLenti-Nef(1 to 115)-(L112A/Y115A)-Flag (M, N) were transfected with siR-Ctrl or siR-hLMP7 for 48 h. Cell lysates were prepared, and the levels of LMP7 and β-actin were measured by Western blotting (K, M). The relative LMP7 band intensity reflects the ratio of LMP7 to β-actin (L, N). (O, P) CD4^+^ Jurkat T cells were infected with pLenti-Flag, pLenti-Nef(1 to 115)-Flag, and pLenti-Nef(1 to 115)-(L112A/Y115A)-Flag, and the expression of Nef(1 to 115)-Flag, Nef(1 to 115)-(L112A/Y115A)-Flag, and β-actin was measured by Western blotting (O). The relative band intensity reflects the ratio of Nef(1 to 115)-Flag or Nef(1 to 115)-(L112A/Y115A)-Flag to β-actin (P). (Q, R) CD4^+^ Jurkat T cells infected with pLenti-Flag, pLenti-Nef(1 to 115)-Flag, or pLenti-Nef(1 to 115)-(L112A/Y115A)-Flag were transfected with siR-Ctrl or siR-hLMP7 for 48 h. Cells were collected and analyzed by flow cytometry after staining with a W6/32 anti-MHC-I-FITC antibody. Cells stained with an IgG isotype antibody served as an MHC-I negative control (Q). MFI was calculated for all W6/32 MHC I-FITC-stained cells (R). The results shown are representative of three independent experiments. The quantitative data represent the mean and standard deviation of three independent experiments. ns, not significant; *, *P* ≤ 0.05; **, *P* ≤ 0.01; ***, *P* ≤ 0.001.

The central task of the iProteasome, executed by the ubiquitin proteasome system (UPS), is to degrade ubiquitylated proteins (Fig. 5A) (38, 39). rhIFN-γ triggers the production of reactive oxygen species (ROS), which engender the oxidative damage of proteins. One of the functions of iProteasome is to target and degrade these defective molecules (38). Therefore, the impact of Nef on the degradation of ubiquitylated proteins by iProteasome under oxidative stress was determined. Cells were transfected with pNef and treated with rhIFN-γ. Defective ubiquitinated proteins were induced by rhIFN-γ, and their level was further enhanced by Nef (Fig. 5B and C). Cells were then infected with HIV-1 (pNL4-3) or Nef-deficient HIV-1 (pNL4-3dNef) and treated with rhIFN-γ. The concentrations of the HIV-1 p24 protein upon stimulation by IFN-γ and infection by Nef-competent virus (HIV-1 NL4-3) or Nef-deficient virus (HIV-1 NL4-3ΔNef) were determined in lysates of CD4^+^ Jurkat cells by enzyme-linked immunosorbent assay (ELISA). These measurements showed that the level of the p24 protein in cells infected by HIV-1 NL4-3ΔNef was higher than that in cells infected by HIV-1 NL4-3 (Fig. 5D). The formation of defective ubiquitinated proteins induced by rhIFN-γ was further enhanced by pNL4-3 but remained unaffected by pNL4-3dNef (Fig. 5E and F). The results demonstrate that Nef inhibits the removal of defective ubiquitinated proteins by attenuating the protein-degrading function of iProteasome.

iProteasomes process cellular proteins into peptides that are loaded onto MHC-I to communicate intracellular protein composition to the immune system (40, 41). The deletion of LMP7 reduces the expression of MHC-I on the cell surface (42). Here, the effects of Nef on MHC-I trafficking and antigen presentation were determined by specifically targeting human LMP7 mRNA through the use of small interfering RNA (siRNA). In CD4^+^ Jurkat T cells, siRNA specifecally targeting human LMP7 mRNA (siR-hLMP7) reduced the expression of LMP7 by 50%, compared to siRNA with scrambled nucleotides (siR-Ctrl) that served as a negative control (Fig. 5G and H). CD4^+^ Jurkat T cells were transfected with siR-hLMP7 or siR-Ctrl, which were negative controls, while cells stained with IgG isotype antibody served as MHC-I negative controls (Fig. 5I). Additionally, the relative mean fluorescence intensity (MFI) of MHC-I was reduced to 60% by Nef in the presence of siR-Ctrl (Fig. 5J), indicating that Nef attenuates the expression of MHC-I on the cell surface. When Nef was stably expressed, the relative MFI was increased by 70% in the presence of siR-hLMP7 (Fig. 5J), suggesting that LMP7 contributes to Nef-mediated attenuation of the expression of MHC-I on the cell surface. Interestingly, the relative MFI was reduced to 90% in the presence of siR-hLMP7 (Fig. 5J), implying MHC-I trafficking attenuation by siR-hLMP7. To assess the relevance of Nef amino acid residues L112 and Y115 for the expression of MHC-I on the cell surface, a cell line stably expressing a Nef double mutant was established (Fig. 5J). In CD4^+^ Jurkat T cells stably expressing Nef(1 to 115) and Nef(1 to 115)-(L112A/Y115A), the expression of LMP7 decreased to approximately 49% and 41%, respectively, after transfection with siR-hLMP7 (Fig. 5K to N). Additionally, the expression of Nef(1 to 115) and Nef(1 to 115)-(L112A/Y115A) was comparable in both cell lines (Fig. 5O and P). The relative MFI of MHC-I was significantly reduced to about 56% by Nef (1 to 115) in the presence of siR-Ctrl, while the MFI increased to 73% when residues L112 and Y115 were mutated to alanine (Fig. 5Q and R). Thus, these data demonstrate that MHC-I trafficking is repressed by Nef and attenuated by siR-hLMP7, implying the involvement of LMP7 in Nef-mediated attenuation of MHC-I trafficking. Moreover, the residues L112 and Y115 of Nef are necessary for this inhibitory activity.

### Nef inhibits the MHC-I antigen presentation pathway by hijacking LMP7.

**FIG 6:**
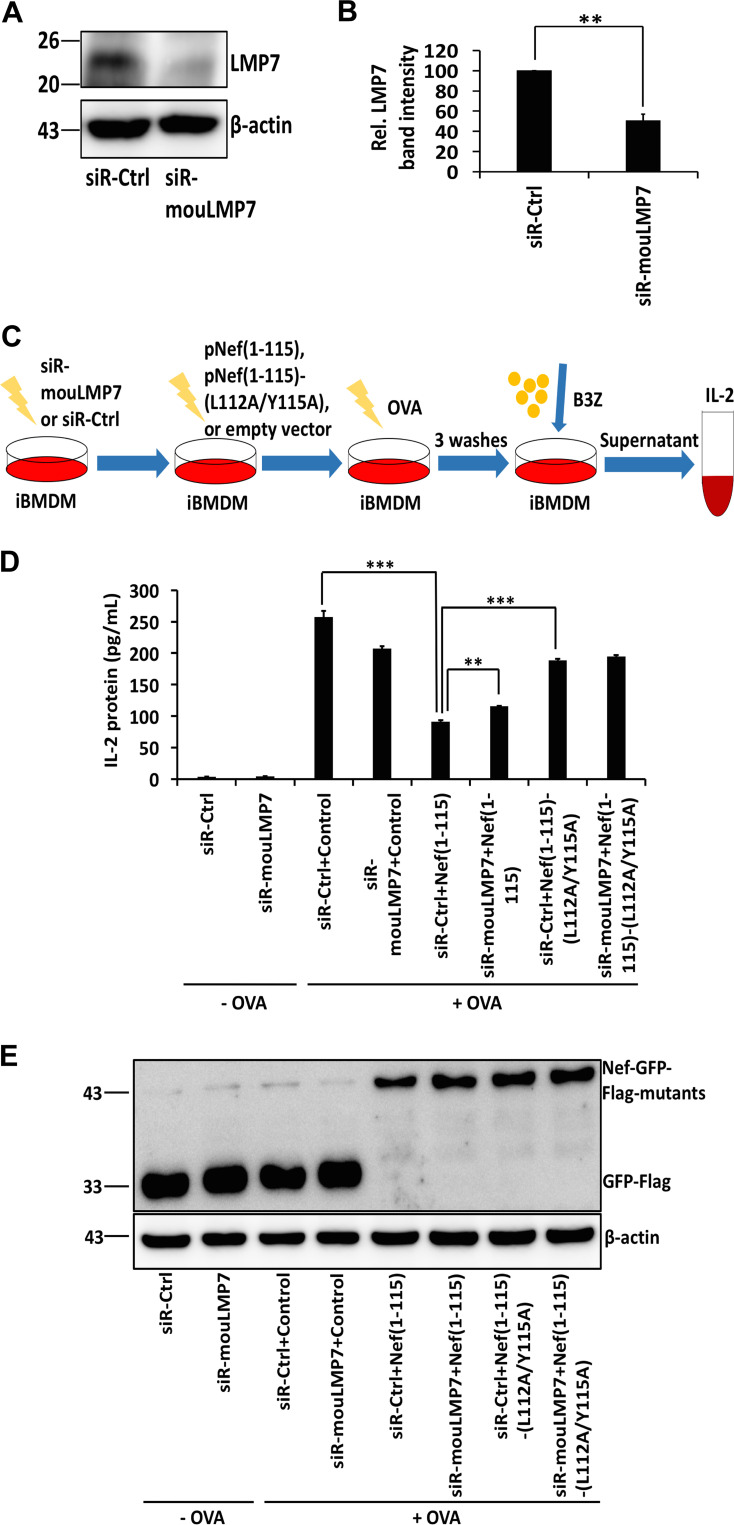
Nef inhibits the MHC-I antigen presentation pathway by hijacking LMP7. (A, B) iBMDM cells were transfected with siR-Ctrl or siR-mouLMP7 for 48 h. Cell lysates were prepared, and the levels of LMP7 and β-actin were measured by Western blotting (A). The intensity of protein bands was quantified; the relative LMP7 band intensity reflects the ratio of LMP7 to β-actin (B). (C) Flow chart of OVA-specific antigen presentation assay. (D, E) iBMDM cells were transfected with siR-Ctrl or siR-mouLMP7 for 24 h and then with an empty vector, pNef(1 to 115)-GFP-Flag, or pNef(1 to 115)-(L112A/Y115A)-GFP-Flag for 24 h. IL-2 secreted by B3Z cells was measured using ELISA. Cells untreated with OVA were considered a negative control (D). The expression of Nef-GFP-Flag mutants and β-actin was measured by Western blotting (E). The results shown are representative of three independent experiments. The quantitative data represent the mean and standard deviation of three independent experiments. ns, not significant; *, *P* ≤ 0.05; **, *P* ≤ 0.01; *** *P* ≤ 0.001.

**FIG 7:**
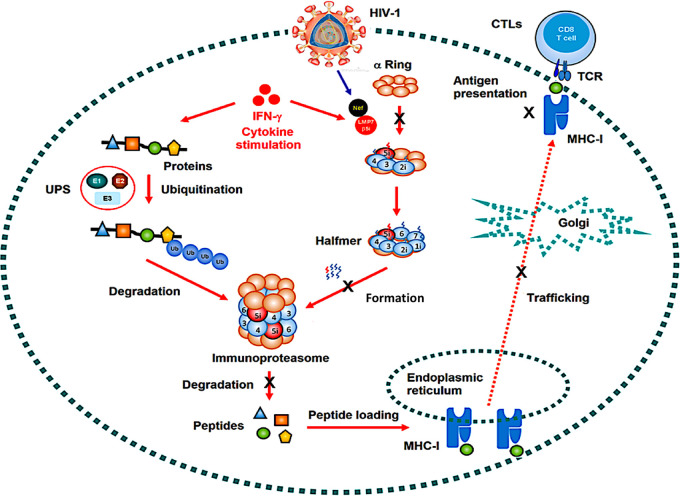
A proposed mechanism by which Nef represses the functions of immunoproteasome and MHC-I. The ubiquitin proteasome system (UPS) is the predominant system responsible for the degradation of 80% cellular proteins. Proteins are targeted for proteasomal degradation via the covalent attachment of ubiquitin. Ubiquitination occurs through the following three enzymes: a ubiquitin-activating enzyme (E1), a ubiquitin-conjugating enzyme (E2), and a ubiquitin-protein ligase (E3). Upon stimulation by IFN-γ, the immunoproteasome becomes the key protein degradation machinery. iProteasome is a highly complex molecular assembly consisting of various components, including the 20S core particle. The 20S core particle of iProteasome has a mass of 700 kDa and comprises 28 protein subunits stacked in 4 homologous rings of 7 subunits, with each forming a hollow cylindrical structure. The two inner rings are each formed by seven β subunits (β1i to 7i) and are enclosed by the two outer rings assembled from seven α subunits (α1 to 7). The proteolytic chamber is formed by the β rings, which harbor the three catalytically active subunits β1i, β2i, and β5i that exhibit caspase-like (CL), trypsin-like (TL), and chymotrypsin-like (ChTL) activity, respectively. iProteasome-degraded products are loaded onto major histocompatibility complex class I (MHC-I), regulating immune responses by inducing cytotoxic-T-lymphocytes (CTLs). However, when HIV-1 viruses infect the host cells, HIV-1 Nef interacts with LMP7, attenuating the formation of iProteasome and its protein degradation function and repressing the trafficking and antigen presentation activity of MHC-I.

iProteasomes mediate immune responses by an efficient generation of peptides for antigen presentation by MHC-I, providing an effective surveillance system of antigen recognition when the host is invaded by pathogens. The initial step in the MHC-I signaling pathway consists of degrading the antigenic proteins into peptides and loading them onto the peptide loading complex (PLC), a process in which iProteasomes play a vital role. MHC-I molecules present a diverse array of antigenic peptides (immunopeptidome) to circulating CTLs. Here, the biological effect of Nef on MHC-I function was determined using an antigen-presenting cell (APC) system (43, 44). An ovalbumin (OVA)-specific antigen presentation assay was performed, in which immortalized bone marrow-derived macrophages (iBMDMs) were transfected with mouse siRNA siR-mouLMP7 or control siRNA siR-Ctrl (Fig. 6A). In comparison with siR-Ctrl, the expression of LMP7 in iBMDMs was reduced to 50% in the presence of siR-mouLMP7 in the presence of siR-Ctrl (Fig. 6B). Next, iBMDM cells were transfected with pNef(1 to 115)-GFP-Flag, pNef(1 to 115)-(L112A/Y115A)-GFP-Flag, or an empty vector; incubated with OVA; and cocultured with mouse CD8^+^ T B3Z cells that recognize the OVA-generated peptide and secrete interleukin-2 (IL-2), an indicator of antigen presentation (Fig. 6C). The secretion of IL-2 was stimulated by OVA in the presence of siR-Ctrl (from 3 pg/ml to 257 pg/ml) or in the presence of siR-mouLMP7 (from 4 pg/ml to 207 pg/ml). However, the induction of IL-2 was inhibited by Nef (1 to 115) in the presence of siR-Ctrl (from 257 pg/ml to 91 pg/ml), while this inhibition by Nef (1 to 115) was significantly impaired in the presence of siR-mouLMP7 (from 91 pg/ml to 115 pg/ml). Moreover, Nef carrying mutations L112A and Y115A increased the secretion of IL-2 in the presence of siR-mouLMP7 (from 91 pg/ml to 188 pg/ml) (Fig. 6D and E). These results indicate that Nef represses antigen presentation by MHC-I by an LMP7-dependent mechanism and residues L112 and Y115 are also essential for the inhibitory activity of Nef. Together, the findings of this study reveal a distinct mechanism by which HIV-1 Nef interacts with LMP7 to promote immune evasion. This novel mechanism relies on the suppression of iProteasome formation and protein degradation and inhibition of MHC-I trafficking and antigen-presentation activity (Fig. 7).

## DISCUSSION

Immunoproteasome activation plays a crucial role in immune responses. This study demonstrated that HIV-1 Nef interacts with LMP7, a key factor in the assembly of iProteasomes (45). Nef attenuates iProteasome formation, promoting evasion of the host immune system. An increasing amount of evidence points to the essential function of LMP7 in inflammatory diseases. Inflammatory disorders are linked to mutations in the LMP7 gene, and pharmacological inhibition of LMP7 generates an anti-inflammatory effect in experimental models of inflammation (46, 47). In addition to its function in immune responses, LMP7 contributes to the management of protein homeostasis under oxidative stress and LMP7 deficiency leads to the formation of intracellular protein aggregates and autoimmune encephalomyelitis (34, 48).

The formation of the iProteasome is an intricate, strictly regulated multistep process that comprises the assembly of an immature 16S precursor, the cleavage of prosequences from protein subunits, and the formation of a mature 20S core particle (33, 34). The β ring of the proteasome is formed on top of the assembled α ring, and the addition adding of β subunits follows a strict sequence (45). The 20S core particle of the proteasome contains three active subunits with caspase-, trypsin-, and chymotrypsin-like activities that are replaced by related proteases LMP2, MECL1, and LMP7 in the iProteasome (49). This substitution renders iProteasomes more efficient at generating peptides for antigen presentation (50). The present work demonstrated that Nef interacts with LMP7 on the ER membrane, attenuating the incorporation of LMP7 into iProteasome, and thereby inhibiting assembly of the iProteasome. Interestingly, both proLMP7-GFP and matLMP7-GFP were able to pull down a larger amount of Nef than proLMP7. The fact that matLMP7 pulled down less Nef than proLMP7 raises the possibility that GFP fused to either full-length or mature domain of LMP7 stabilizes their structures and enhances their binding to Nef. Moreover, Nef represses the degradation of defective proteins by the iProteasome upon IFN stimulation, indicating that Nef is implicated in protein ubiquitination under oxidative stress.

The iProteasome mediates immune responses by efficiently generating peptides for antigen presentation of MHC-I, which is an efficient surveillance system that present antigens when hosts are invaded by pathogens (51). The beginning step in the MHC-I signaling pathway is processing the antigenic proteins into peptides and loading them onto PLCs, in which the iProteasomes play a vital role (52). MHC-I molecules present a diverse array of antigenic peptides (immunopeptidome) to circulating TCLs (53). Interestingly, Nef represses the trafficking and antigen presentation activities of MHC-I.

Nef represses the trafficking and antigen presentation activities of MHC-I and, therefore, is able to counteract immune responses of the host (23–27). This study identified a distinct mechanism by which HIV-1 promotes immune evasion by attenuating the functions of the iProteasome and MHC-I. This mechanism involves the interaction of Nef with LMP7 on the ER membrane and leads to the attenuation of LMP7 incorporation into the iProteasome, inhibition of the formation of iProteasome and its protein degradation function, and repression of the trafficking and antigen presentation by MHC-I.

## MATERIALS AND METHODS

### Cells and cell culture.

Human embryonic kidney (HEK)293T cells, epithelial cervical adenocarcinoma (HeLa) cells, and immortalized bone marrow-derived macrophage (iBMDM) cells were obtained from the China Center for Type Culture Collection (CCTCC) (Wuhan, China). Cells were cultured in Dulbecco’s modified Eagle’s medium (DMEM) (Gibco, Grand Island, NY, USA) containing 5% fetal bovine serum (FBS) (Gibco, Gaithersburg, MD, USA), 1% penicillin, and 1% streptomycin at 37°C in the presence of 5% CO_2_. CD4^+^ Jurkat T cells were obtained from CCTCC, and B3Z cell hybridoma was a gift of Nilabh Shastri of the University of California at Berkeley. Jurkat and hybridoma cells were cultured in RPMI 1640 medium (Gibco) containing 10% FBS, 1% penicillin, and 1% streptomycin at 37°C in the presence of 5% CO_2_. Plasmid transfections were performed using Lipofectamine 2000 (Invitrogen, Carlsbad, CA, USA) according to the manufacturer’s instructions.

### Reagents.

Recombinant human IFN-γ (rhIFN-γ) was purchased from Peprotech (Rocky Hill, NJ, USA). ER-Tracker Red dyes were purchased from Invitrogen. Egg white ovalbumin (OVA) was purchased from Sangon Biotech (Shanghai, China). Sulfosuccinimidyl-6-(biotinamido)-6-hexanamido hexanoate (NHS-LC-LC)-biotin was purchased from Thermo Fisher Scientific (Waltham, MA, USA).

### Virus infection.

A wild-type HIV-1 plasmid (pNL4-3) was obtained from the NIH (Rockville, MD, USA) and used to generate the Nef-deficient HIV-1 plasmid (pNL4-3dNef). pNL4-3 and pNL4-3dNef were transfected into HEK293T cells using polyethylenimine (PEI) transfection reagents (Polysciences, PA, USA). At 48 h posttransfection, cell culture media were collected and the cell debris was removed by centrifugation. Viral titer was determine using the p24 enzyme-linked immunosorbent assay (ELISA) (R&D Systems, MN, USA). CD4^+^ Jurkat T cells were incubated with NL4-3 or NL4-3dNef viruses for 2 h at a multiplicity of infection (MOI) of 10 ng p24 per 10^6^ cells. The cells were then washed, and a fresh culture medium was added. The cells and supernatants were collected at the indicated time for further analyses.

### Stable cell lines.

Flag-tagged Nef, Nef(1 to 115), or Nef(1 to 115)-(L112A/Y115A) genes were constructed into the pLenti vector (Invitrogen). HEK293T cells were cotransfected with pLenti-Nef, pNef(1 to 115), and pNef(1 to 115)-(L112A/Y115A) plasmids or pLenti empty vectors and pLP1, pLP2, and pLP/VSVG plasmids (Invitrogen) using PEI transfection reagents. Culture medium containing lentiviruses was collected 2 days after the transfection. CD4^+^ Jurkat T cells were infected with lentiviruses containing Nef, Nef(1 to 115), Nef(1 to 115)-(L112A/Y115A), or an empty vector. Stable Nef-, Nef(1 to 115)-, or Nef(1 to 115)-(L112A/Y115A)-expressing cells were selected with 2.5 μg/ml puromycin.

### Protein polyubiquitylation.

HeLa cells were incubated with 100 U/ml rhIFN-γ 1 day after transfection with Nef plasmids or empty vectors. CD4^+^ Jurkat cells were incubated with 100 U/ml rhIFN-γ 2 h after infection with wild-type NL4-3 or Nef-deficient NL4-3dNef virus. The cells were harvested and lysed for Western blotting at the indicated time.

### Plasmid construction.

The full-length and truncated Nef were inserted into pCAGGS vectors (BCCM/LMBP Plasmid Collection, Ghent, Belgium) with a hemagglutinin (HA) tag linked to the C-terminal insert. Nef mutants were constructed into pCAGGS vectors using an overlapping PCR, and the putative amino acid residues were mutated to alanine. Alternatively, full-length Nef with the GFP gene fused to its N terminus was inserted into the pLenti vector (Invitrogen). Full-length and truncated LMP7 were inserted into pcDNA3.1 vectors (Invitrogen) with a 3× Flag tag linked to the N-terminal insert. Additionally, full-length LMP7 was inserted into the pCMV vector (Clontech, Fremont, CA, USA) with a Myc tag linked to the N-terminal insert. For the yeast two-hybrid assays, Nef was inserted into the pGBKT7 vector and LMP7 into the pGADT7 vector (Clontech). To generate pNL4-3dNef, the XhoI restriction enzyme site in pNL4-3 was digested, and a stop codon was inserted.

### Confocal microscopy.

HeLa cells were seeded onto 15-mm glass-bottom dishes (Nest Scientific, NJ, USA). One day later, the cells were transfected using 1 μg of plasmids per dish. At 24 h posttransfection, cells were washed 3 times with phosphate buffered saline (PBS) and fixed with 1% formaldehyde in PBS for 30 min. Subsequently, the cells were washed 3 times with PBS, permeabilized for 30 min with 0.1% saponin in PBS supplemented with containing 1% bovine serum albumin (BSA), and incubated with anti-HA or anti-Flag (Sigma-Aldrich, St. Louis, MO, USA) for 1 h. Cells were then washed 3 times with permeabilization buffer and incubated with secondary antibodies for 1 h. Finally, cells were washed 3 times with PBS, stained with the endoplasmic reticulum (ER)-Tracker Red dye (Invitrogen) for 30 min, and washed again 3 times in PBS. The specimens were viewed using a FluoView FV1000 (Olympus, Tokyo, Japan) confocal microscope.

### Recombinant protein expression and purification.

Nef and LMP7 genes were cloned into pGEX-6P-1 (GE, Boston, MA, USA) and pET-28a (Novagen, Madison, WI, USA) plasmids, respectively. The pGEX-6P-1 empty plasmid, pEGX-6P-1-Nef plasmid, and pET-28a-LMP7 plasmid were transformed into BL21(DE3) competent cells (TransGen, Beijing, China). The cells were grown in LB buffer with 0.1 mM isopropyl-β-d-thiogalactopyranoside (IPTG) at 30°C or 16 h and harvested by centrifugation at 3,000 × *g* for 10 min. Cell pellets were resuspended in PBS, frozen and thawed 3 times, and sonicated for 10 min (pulse on for 5 s and pulse off for 5 s) on ice. Sonicated samples were centrifuged at 12,000 rpm for 10 min, the supernatants were collected, and proteins were purified using the NGC Scout 10 system (Bio-Rad, Hercules, CA, USA) according to the manufacturer’s protocol.

### *In vitro* GST pulldown assay.

The LMP7 protein, expressed in bacterial cells, was mixed with purified GST or GST-Nef proteins in radioimmunoprecipitation assay (RIPA) buffer (50 mM Tris-HCl [pH 7.4] 150 mM NaCl, 0.25% deoxycholate, and 1% NP-40). The samples were then combined with recombinant protein G-Sepharose 4B beads (Thermo Fisher Scientific) and 1 μl of anti-Flag antibody (Sigma-Aldrich) and incubated overnight at 4°C. The beads were washed 4 times with RIPA buffer and mixed with loading buffer, and the proteins were separated by sodium dodecyl sulfate-polyacrylamide gel electrophoresis (SDS-PAGE) and analyzed by Western blotting.

### Biolayer interferometry (BLI) assay.

The purified LMP7 protein from bacteria was linked with NHS-LC-LC-biotin (Thermo Fisher Scientific) at a 1:3 molar ratio by incubating them at room temperature for 1 h. Unreacted biotin was removed using the Zeba spin desalting columns (Thermo Fisher Scientific). The streptavidin biosensors (ForteBio, Menlo Park, CA, USA) were sequentially dipped into wells of black 96-well plates (Greiner, Kremsmünster, Austria) containing: PBS; LMP7; PBS; GST or Nef-GST; PBS. All association and dissociation curves were fitted to a 1:1 binding model.

### Proteasome purification.

HeLa cells were seeded in 10-cm dishes 1 day before transfections. The cells were transfected at 50% to 80% confluence by adding 10 μg Nef-HA or empty vector plasmids per dish and mixing with 20 μl PEI transfection reagents. At 24 h after the transfection, the cells were treated with 100 U/ml IFN-γ (Peprotech, Rocky Hill, NJ, USA) for 24 h. Cells were then washed with prechilled PBS, trypsinized, and lysed in 1 ml of buffer A (50 mM Tris-HCl [pH 7.5], 250 mM sucrose, and 150 mM NaCl) by 3 cycles of freezing and thawing. The lysates were preclarified by centrifugation at 1,000 × *g* for 5 min at 4°C, and the supernatant was clarified again at 10,000 × *g* for 20 min at 4°C. A 20-μl aliquot of the supernatant of each sample was saved and stored at 4°C until further analysis. The remaining part of each sample was centrifuged in buffer A at 100,000 × *g* for 1 h at 16°C. The pellets were discarded, and the supernatant was centrifuged again at 100,000 g for 5 h at 16°C. The pellets were dissolved in 20 μl buffer B (50 mM Tris-HCl [pH 7.5], 150 mM NaCl, and 15% glycerol) and analyzed by Western blotting.

### Western blotting and immunoprecipitation.

HEK293T cells were plated in 6-cm dishes and, after 1 day, were transfected by mixing with 4 μg of plasmids and 8 μl of PEI transfection reagents per plate. At 24 h posttransfection, cells were lysed in RIPA buffer containing 1 mM phenylmethylsulfonyl fluoride (PMSF) (Sigma-Aldrich). Cell lysates were clarified by centrifugation at 12,000 rpm for 10 min, mixed with SDS-PAGE loading buffer (50 mM Tris-HCl [pH 6.8], 2% SDS, 10% glycerol, 0.1% bromophenol blue, and 1% 2-mercaptoethanol), and heated at 95°C for 10 min. The samples were loaded onto 12% SDS-PAGE gels and run at 120 V for 2 h. Separated proteins were transferred to polyvinylidene difluoride (PVDF) membranes (GE) at 70 V for 2 h. The membranes were blocked with 2% nonfat milk in PBS containing 0.1% Tween 20 (PBST) for 30 min at room temperature. After being washed 3 times with PBST, membranes were incubated with primary antibodies overnight, washed 3 times with PBST, and incubated with secondary antibodies for 1 h. After 6 washes with PBST, the membranes were incubated with the Clarity Western ECL substrate (Bio-Rad) and scanned using a LAS-4000 system (FujiFilm, Tokyo, Japan). Alternatively, cell lysate supernatants were mixed with antibodies and recombinant protein G-Sepharose 4B beads (Thermo Fisher) and incubated at 4°C overnight. The beads were washed 4 times with RIPA buffer, mixed with SDS-PAGE loading buffer, and subjected to SDS-PAGE.

### Flow cytometry.

CD4^+^ Jurkat T cells stably expressing empty vector, Nef-Flag, Nef(1 to 115)-Flag, or Nef(1 to 115)-(L112A/Y115A)-Flag were seeded in 6-well plates 1 day before transfection. The cells were transfected with 50 nM LMP7 or control siRNA by mixing with 7 μl of INTERFERin transfection reagents (Polyplus-transfection, Illkirch, France). Twenty-four hours after the transfection by siRNA, cells were treated with 100 U/ml rhIFN-γ for 24 h. The cells were collected, fixed in 1% formaldehyde in PBS for 10 min, and incubated with blocking buffer (1% BSA in PBS) for 30 min and then with W6/32 APC or isotype control antibodies for 1 h. After the aggregates were filtered out, the cells were analyzed using the Cytoflex instrument (Beckman, Brea, CA, USA); 50,000 events were collected for each sample.

### Antigen presentation assay.

iBMDM cells were seeded in 24-well plates. After the cells became attached and reached 50% confluence, they were transfected with 500 ng of GFP, Nef(1 to 115)-GFP-Flag, or Nef(1 to 115)-(L112A/Y115A)-GFP-Flag plasmids for 15 h. After cells were washed 3 times with PBS, Opti-MEM I reduced serum medium (Thermo Fisher) supplemented with 10 mg/ml OVA was added for 6 h. The cells were then washed 3 times with PBS and cocultured with B3Z cells for 24 h. The medium was collected, centrifuged at 12,000 rpm for 10 min to discard the cell debris, and frozen at –80°C until analysis. The IL-2 concentration was measured using the mouse IL-2 ELISA set (BD Biosciences, Franklin Lakes, NJ, USA) according to the manufacturer’s protocol.

### Yeast two-hybrid assay.

Nef and LMP7 genes were inserted into pGBKT7 and pGADT7 vectors (Clontech), respectively. Y2HGold and Y187 yeast strains (Clontech) were transformed with pGBKT7-Nef and pGADT7-LMP7 plasmids and grown at 30°C for 3 days. Y2HGold and Y187 yeasts were picked and mixed together to grow overnight in a yeast peptone dextrose adenine (YPDA) broth at 200 rpm and 30°C. The mated culture was plated onto double dropout (DDO) (−Leu/−Trp) agar plates and incubated at 30°C for 3 days. Colonies were picked and streaked on quadruple dropout (QDO) (−Ade/−His/−Leu/−Trp) and X-α-Gal agar plates and grown at 30°C for 3 days.

### Statistics.

All experiments were repeated at least three times with similar results. All results are expressed as the mean ± standard deviation (SD). Statistical analysis was performed using the two-tailed *t* test (GraphPad Prism 5). Differences were considered statistically significant at the following values: *, *P* ≤ 0.05, **, *P* ≤ 0.01, and ***P ≤ 0.001. ns, not significant.
